# Contribution of the Major ND10 Proteins PML, hDaxx and Sp100 to the Regulation of Human Cytomegalovirus Latency and Lytic Replication in the Monocytic Cell Line THP-1

**DOI:** 10.3390/v7062751

**Published:** 2015-06-05

**Authors:** Nadine Wagenknecht, Nina Reuter, Myriam Scherer, Anna Reichel, Regina Müller, Thomas Stamminger

**Affiliations:** Institute of Clinical and Molecular Virology, Friedrich-Alexander-Universität Erlangen-Nürnberg, Schlossgarten 4, 91054 Erlangen, Germany; E-Mails: nadine.wagenknecht@viro.med.uni-erlangen.de (N.W.); nina.reuter@viro.med.uni-erlangen.de (N.R.); myriam.scherer@viro.med.uni-erlangen.de (M.S.); aareiche@viro.med.uni-erlangen.de (A.R.); regina.mueller@viro.med.uni-erlangen.de (R.M.)

**Keywords:** cytomegalovirus, latency, intrinsic immunity, restriction factor, ND10, PML

## Abstract

Promyelocytic leukemia nuclear bodies, also termed nuclear domain 10 (ND10), have emerged as nuclear protein accumulations mediating an intrinsic cellular defense against viral infections via chromatin-based mechanisms, however, their contribution to the control of herpesviral latency is still controversial. In this study, we utilized the monocytic cell line THP-1 as an *in vitro* latency model for human cytomegalovirus infection (HCMV). Characterization of THP-1 cells by immunofluorescence and Western blot analysis confirmed the expression of all major ND10 components. THP-1 cells with a stable, individual knockdown of PML, hDaxx or Sp100 were generated. Importantly, depletion of the major ND10 proteins did not prevent the terminal cellular differentiation of THP-1 monocytes. After construction of a recombinant, endotheliotropic human cytomegalovirus expressing IE2-EYFP, we investigated whether the depletion of ND10 proteins affects the onset of viral IE gene expression. While after infection of differentiated, THP-1-derived macrophages as well as during differentiation-induced reactivation from latency an increase in the number of IE-expressing cells was readily detectable in the absence of the major ND10 proteins, no effect was observed in non-differentiated monocytes. We conclude that PML, hDaxx and Sp100 primarily act as cellular restriction factors during lytic HCMV replication and during the dynamic process of reactivation but do not serve as key determinants for the establishment of HCMV latency.

## 1. Introduction

Herpesviral latency is defined as a stage in which the viral genome exists as an episome within the nucleoplasm in the absence of lytic gene expression and production of infectious virus [1]. It is now generally believed that human cytomegalovirus (HCMV) is able to establish latency in hematopoietic progenitor cells of the myeloid lineage and differentiation of these cells to a mature phenotype is linked to viral reactivation [2,3,4,5,6,7]. The ability of HCMV to establish lifelong infection in humans and to reactivate from latency in immunocompromised individuals leading to devastating disease underscores the importance of understanding the mechanisms that regulate latency and reactivation. It is thought that expression of viral immediate early (IE) proteins IE1 and IE2, which initiate the lytic phase, must be suppressed during latency [5]. Silencing of viral IE gene expression is achieved by a repressive chromatin structure around the viral major IE promoter (MIEP) resulting from specific post-translational modifications of histones [5]. As shown for CD34^+^ cells, the heterochromatin protein-1 (HP-1) was found to be associated with the MIEP and tri-methylation of histone H3 (lysine 9 and 27) as well as the absence of histone acetylation on histone H4 could be detected, thus representing a typical repressive chromatin phenotype [8]. This chromatin phenotype is maintained in the monocytes derived from precursor cells [9]. Only upon further cellular differentiation robust IE gene expression can be detected allowing for reactivation of infectious virus [10].

Interestingly, silencing of viral DNA genomes through chromatinization and epigenetic histone modification seems to constitute a general mechanism that is not only used to establish herpesviral latency but also serves as an intrinsic immune mechanism of the cell to shut off incoming viral genomes [11,12]. In particular, we as well as others could demonstrate that proteins accumulating at a specific subnuclear structure, termed nuclear domain (ND) 10, are able to silence viral IE gene expression and this involves a recruitment of transcriptionally repressive chromatin marks around the MIEP [13,14,15,16]. ND10, alternatively termed promyelocytic leukemia protein (PML) nuclear bodies, are dynamic macromolecular structures that are defined by the presence of the major components PML, hDaxx, and Sp100, which accumulate in distinct foci within the interchromosomal space of the nucleus [17]. The PML protein serves as the key organizer of ND10 as it is responsible for the recruitment of a large number of proteins to this matrix-associated structure which share the capability to be posttranslationally modified by SUMO [18]. In fact, both covalent as well as non-covalent SUMO interactions are regarded to constitute the basis for PML body formation since only SUMOylated PML is capable of orchestrating ND10 structures [19,20]. For instance, hDaxx has been shown to bind to SUMOylated PML via a critical SUMO interaction motif (SIM) and this interaction appears to be regulated via additional posttranslational modifications such as phosphorylation and acetylation [21,22,23]. Several recent studies demonstrated that the major ND10 components PML, hDaxx and Sp100 independently contribute to the silencing of herpesviral gene expression, so that their individual activities combine to maximize the antiviral effect [24,25,26]. However, this cellular restriction instituted by ND10 factors is amenable to viral antagonization. For instance, during HCMV infection, the tegument protein pp71 induces a proteasomal degradation of hDaxx, while the IE1 protein disperses ND10 accumulations by deSUMOylating its scaffold factor PML [27,28,29].

So far, ND10 functions have mainly been investigated in models of productive HCMV infection. However, since the viral MIEP adopts a similarly silenced chromatin structure in latently infected cells, it was speculated that individual ND10 proteins might contribute to the establishment of viral latency. A previous study performed by Saffert and Kalejta suggested that IE gene expression is silenced by hDaxx in latent infections [30]. More precisely, by using two *in vitro* latency models, NT2 and THP-1 cells, they could show that pp71 failed to inactivate the hDaxx protein since pp71 could only be detected in the cytoplasm and was thus localized in another cell compartment than hDaxx. These findings were extended by the observation that hDaxx also contributes to the silencing of IE gene expression in primary human CD34^+^ cells [31]. However, it was shown that only viral IE gene expression of a laboratory-adapted HCMV strain, but not of a clinical strain, could be rescued by knockdown of hDaxx in this system [31]. In a third experimental setting, the Kalejta group could show that in two CD34^+^ myeloblastic cell lines, KG-1 and Kasumi-3, IE genes were silenced similar to primary CD34^+^ cells, however, in contrast to primary CD34+ cells or THP-1 cells viral IE gene expression could not be induced by the presence of HDAC inhibitors [32]. In contrast, the group of Sinclair proposed little involvement of the hDaxx protein in the regulation of the viral MIEP in latently infected cells since knockdown of hDaxx in undifferentiated NT2 cells did not permit IE gene expression [33]. Thus, the contribution of hDaxx for the establishment of HCMV latency is still controversial and, hence, the aim of this study was to further clarify the relevance of individual ND10 factors for this process. Importantly, we wished to perform a comparative analysis of the major ND10 proteins, PML, hDaxx and Sp100 in a system that allows assessment of the role of individual restriction factors for the control of latency and lytic replication in parallel.

For this, the extensively investigated *in vitro* latency model of THP-1 monocytes was used: While HCMV infection of non-differentiated THP-1 cells results in latent carriage of the viral genome, the cells become permissive for HCMV lytic infection after induction of cellular differentiation by treatment with phorbol 12-myristate 13-acetate (PMA) [34,35,36,37,38,39]. We constructed a recombinant cytomegalovirus expressing IE2 in fusion with EYFP, which served as a sensitive marker virus for flow cytometry-based detection of lytic viral gene expression. After infection of non-differentiated THP-1 cells, we observed that an shRNA-mediated depletion of PML, hDaxx or Sp100 did not affect the initiation of viral IE gene expression. In contrast, after differentiation towards a macrophage-like phenotype, the lack of major ND10 proteins significantly increased the number of cells starting the viral gene expression program. We conclude that PML, Sp100 and hDaxx primarily act as cellular restriction factors that do not serve as key determinants for the establishment of HCMV latency, but may affect the reactivation of HCMV from latency.

## 2. Materials and Methods

### 2.1. Cell Culture and Virus Infection

HEK293T cells were cultivated in Dulbecco’s minimal essential medium (DMEM) containing 10% fetal calf serum at 37 °C and 5% CO_2_. Primary human foreskin fibroblasts (HFFs) were prepared from human foreskin tissue, as described previously [40], and were maintained in Eagle’s minimal essential medium (GIBCO/BRL, Eggenstein, Germany) supplemented with 5% fetal calf serum. The monocytic cell line THP-1 was maintained in Roswell Park Memorial Institute medium (RPMI 1640) supplemented with 10% fetal calf serum at 37 °C and 5% CO_2_. Differentiation of THP-1 cells was induced by treatment with 50 nM phorbol 12-myristate 13-acetate (PMA) for 24 to 72 h.

Infection experiments were performed with the HCMV strain TB40E and the recombinant viruses TB40E/IE1-mCherry and TB40E/IE2-EYFP. Titration of the viral stocks was performed by IE1p72 fluorescence [41]. Briefly, HFFs (80,000 cells) in 0.5 mL medium were seeded into 24-well dishes and infected the next day with 500 μL of various dilutions (1:5 to 1:10^5^) of viral supernatants. At 24 hpi, cells were fixed with methanol and stained with monoclonal antibody p63-27 which is directed against IE1p72 [42]. Finally, the number of IE1-positive cells was determined and was used to calculate viral titers indicated in IE protein-forming units (IEU) per mL.

For infection of HFFs with wt and recombinant HCMVs, 3.0 × 10^5^ cells were seeded into 6-well dishes. One day later, the medium of HFFs was replaced by 1 mL of infectious cell culture supernatant and after incubation for 2 h, the supernatant was substituted with fresh medium.

Infection of THP-1 monocytes with TB40E/IE2-EYFP was performed by resuspending the cells in the respective amount of viral supernatant for 2 h in standard culture plates followed by two washing steps of the cells with PBS and finally incubation of the infected THP-1 cells in fresh medium overnight. THP-1-derived macrophages were infected in the same way as HFF cells.

### 2.2. Retroviral Transduction and Selection of Stably Transduced Cells

For the generation of THP-1 cells stably expressing the respective siRNAs siC, siPML, siDaxx and siSp100, the Lenti-X shRNA expression system based on vector pLVX-shRNA1 was applied. This system relies on the generation of replication incompetent, VSV-G pseudotyped lentiviruses, which, upon infection of cells, facilitate RNAi-mediated knockdown via the expression of shRNAs in dividing and non-dividing mammalian cells. In a first step, the nucleotide sequences of the shRNAs targeting PML, hDaxx and Sp100, as described in previous studies, were inserted into vector pLVX-shRNA1 via *Bam*HI and *Xho*I [25]. Then, HEK293T cells, which served as a producer cell line for the generation of infectious retroviral particles, were seeded into 10 cm dishes at a density of 5 × 10^6^ cells/dish followed by overnight incubation. The next morning, the cell culture medium was replaced with 5 mL fresh medium without antibiotics. For the generation of lentiviral particles, 3 μg of pLVX-shRNA1-derived vectors were mixed with 36 μL of the ready-to-use Lenti-X HTX packaging mix 2 (Clontech, Palo Alto, CA, USA) in 1.5 mL of medium without antibiotics and FBS. In parallel, 36 μL of lipofectamine 2000 reagent (Invitrogen, Karlsruhe, Germany) were diluted in 1.5 mL of medium without antibiotics and FBS (DMEM, 350 μg/mL L-glutamine) and incubated for 5 min at RT. Next, lipofectamine 2000-containing solutions were added to the diluted DNA, incubated at RT for further 20 min to allow the formation of DNA-lipofectamine complexes. Thereafter, the transfection mixture was evenly distributed over the cells. After overnight incubation at 37 °C, the transfection medium was substituted with fresh medium. Finally, the viral supernatant was harvested at 48 h post transfection, clarified by centrifugation for 15 min at 3000 rpm followed by filtration using a 0.45 μm filter (Millipore, Schwalbach, Germany) to completely remove all cell debris. The viral inocula were either directly used for transduction of THP-1 cells or were stored in 1 mL aliquots at −80 °C. In order to generate cells stably expressing shRNAs, the suspension monocytic cell line THP-1 was transduced with the lentiviral particles in the presence of polybrene followed by puromycin-selection of stably transduced cell populations.

### 2.3. Generation of the Recombinant Viruses TB40E/IE1-mCherry and TB40E/IE2-EYFP

For fusion of mCherry or EYFP to the coding region of IE1 or IE2, respectively, a markerless BAC mutagenesis according to Tischer *et al.* [43] was performed in the *E. coli* strain GS1783 (kindly provided by Karsten Tischer, Berlin, Germany). Linear recombination fragments were generated by amplification of an I-*Sce*I-*aphAI* cassette from plasmid pHM3367 (universal transfer construct based on pEPkan-S, for mCherry fusion) or pHM3366 (universal transfer construct based on pEPkan-S, for EYFP-fusion) via Hot Start PCR with the polymerase Phusion Hot Start (Thermo Fisher Scientific, Waltham, MA, USA) and specific primer pairs (Table 1). *DpnI* was added to digest template DNA and the amplicon was purified from an agarose gel with the Qiagen Gel Extraction Kit (Qiagen, Hilden, Germany). In order to accomplish homologous recombination, PCR fragments were transformed into *E. coli* strain GS1783, which encodes the restriction homing endonuclease I-*Sce*I under an arabinose-inducible promoter and harbors the HCMV BAC TB40E-Bac4 (kindly provided by Christian Sinzger, Ulm, Germany). Positive transformants were identified using agar plates containing chloramphenicol as well as kanamycin. Next, in order to remove the kanamycin cassette, I-*Sce*I was induced by the addition of 1% arabinose followed by a second Red recombination. Finally, bacteria were grown on chloramphenicol and 1% arabinose LB-plates at 32 °C for 1 to 2 days and kanamycin-sensitive colonies were identified by replica plating. BAC DNA was isolated from bacteria using the BAC preparation protocol of the PureLink HiPure Plasmid Maxiprep kit (Invitrogen, Karlsruhe, Germany). The obtained BACs were verified by distinct PCR reactions and subsequent nucleotide sequence analyses as well as restriction fragment length polymorphism analysis (RFLP) as described previously [44]. In order to reconstitute infectious viruses, HFFs were transfected with the obtained BAC DNA using X-tremeGENE transfection reagent (Roche, Mannheim, Germany). Cells were incubated until the appearance of distinct cytopathic changes followed by the preparation of virus stocks. This was done by cultivation of large quantities of the recombinant virus in primary human fibroblasts until a complete cytopathic effect was achieved, followed by centrifugation of the cell culture supernatant to remove cellular debris and storage at −80 °C in aliquots.

**Table 1:** Oligonucleotide primers for generation of recombinant human cytomegalovirus strains.

Name	Sequence in 5′-3′
IE1-mCherry fw	CTGGAGGCAAGAGCACCCACCCTATGGTGACTAGAAGCAAGGCTGACCAGATGGTGAGCAAGGGCGAGGAGGAT
IE1-mCherry rev	TAGTGACGTGGGATCCATAACAGTAACTGATATATATATACAATAGTTTACTTGTACAGCTCGTCCATGCCGCC
IE2-EYFP-fw	TGAGCCTGGCCATCGAGGCAGCCATCCAGGACCTGAGGAACAAGTCTCAGATGGTGAGCAAGGGCGAGGAGCTG
IE2-EYFP-rev	GGGGAATCACTATGTACAAGAGTCCATGTCTCTTTCCAGTTTTTCACTTACTTGTACAGCTCGTCCATGCCGAG

### 2.4. Fluorescence-Activated Cell Sorter (FACS) Analysis

For FACS analysis, 2 × 10^5^ TB40E/IE2-EYFP infected THP-1 monocytes were harvested at the indicated times post infection and cells were washed with FBS-containing buffer (2% FBS and 2 mM EDTA in PBSo (138 mM NaCl, 2.7 mM KCl, 6.5 mM Na_2_HPO_4_, 1.5 mM KH_2_PO_4_)). Then, the cells were fixed by the addition of 2% formaldehyde. Finally, samples were analyzed with the BD LSR II Flow Cytometer (BD Biosciences, Franklin Lakes, NJ, USA) and the results were evaluated with FCS Express V3 (De Novo Software, Los Angeles, CA, USA).

### 2.5. Indirect Immunofluorescence Analysis and Western Blotting

For indirect immunofluorescence analysis, THP-1 monocytes were grown on polylysine-coated coverslips in 6-well dishes (6 × 10^5^ cells/well) followed by fixation with 4% paraformaldehyde for 10 min at room temperature. THP-1-derived macrophages grown on coverslips in 6-well dishes (6 × 10^5^ cells/well) were fixed with 4% paraformaldehyde at the indicated time points after induction of differentiation. Then, cells were washed with PBS three times followed by permeabilization of the cells with 0.2% Triton-X 100 solution on ice for 20 min. After another washing step, cells were incubated with a blocking solution (2 mg/mL Cohn II in PBSo) for 30 min at 37 °C. Next, cells were stained with 500 μL of the respective primary antibody diluted in PBSo-1% FBS for 30 min at 37 °C. The corresponding secondary antibody conjugated to the respective fluorescent dye was likewise diluted in 500 μL of PBSo-1% FBS and incubated with the cells for further 30 min at 37 °C. Finally, cells were mounted by using Vectashield mounting medium plus 4′,6-diamidino-2-phenylindole (DAPI; Vector Laboratories, Burlingame, CA, USA). The samples were analyzed using the Leica TCS SP5 confocal laser scanning microscope (Leica, Wetzlar, Germany) and images were processed using Photoshop. Colocalization analysis was performed by obtaining maximum intensity Z projections of 10 successive slices using LAS AF software (Leica).

For Western blot analysis, extracts from mock- or HCMV-infected cells were prepared in an SDS-PAGE loading buffer, separated by SDS-polyacrylamide gel electrophoresis (PAGE) on 8%–12.5% polyacrylamide gels and transferred to nitrocellulose membranes. Chemiluminescence was detected according to the manufacturer’s protocol (ECL Western blot detection kit; Amersham Pharmacia Biotech).

### 2.6. Antibodies

Monoclonal antibody (MAb) p63-27 for detection of IE1, anti-pHM178 antisera directed against IE2 and MAb 28-4 recognizing the major capsid protein (MCP) have been described elsewhere [42,45,46]. MAb-UL44 BS 510, for detection of the viral polymerase processivity factor pUL44, was kindly provided by Bodo Plachter (Institute of Virology, Mainz, Germany). For detection of beta-actin, the monoclonal antibody AC-15 was purchased from Sigma-Aldrich (Deisenhofen, Germany). PML was detected with the rabbit polyclonal antibodies A301-167A and A301-168A (Bethyl Laboratories, Montgomery, TX, USA) and the rabbit polyclonal antibody H-238 (Santa Cruz Biotechnology, Santa Cruz, CA, USA), Sp100 by using the rabbit polyclonal antiserum GH3 (a kind gift from Hans Will, University of Hamburg, Hamburg, Germany) and the mouse polyclonal antibody H0006672-B01 (Abnova, Heidelberg, Germany). HDaxx was detected by applying a rabbit monoclonal antibody from Epitomics (Burlingame, CA, USA) and the goat polyclonal antibody C20 (Santa Cruz Biotechnology, Santa Cruz, CA, USA). For Western Blot analysis, horseradish peroxidase-conjugated anti-mouse and anti-rabbit secondary antibodies were purchased from Dianova (Hamburg, Germany) and for indirect immunofluorescence analysis, Alexa Fluor 488- and Alexa Fluor 555-conjugated secondary antibodies were obtained from Molecular Probes (Karlsruhe, Germany).

## 3. Results

### 3.1. Analysis of the Subnuclear Localization and the Expression Pattern of the Major ND10 Proteins PML, Sp100 and hDaxx in Undifferentiated THP-1 Monocytes and THP-1 Derived Macrophages

**Figure 1:**
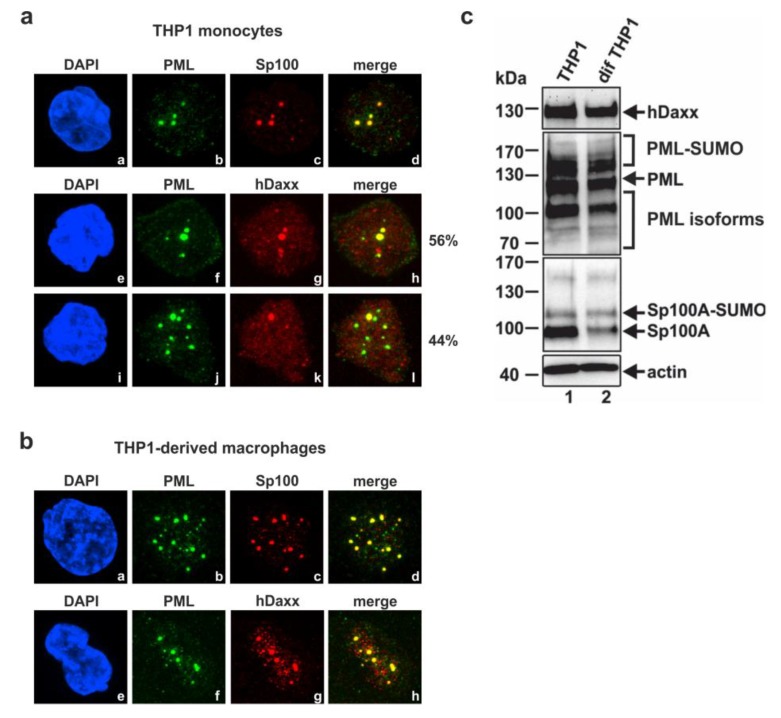
(**a**) Subnuclear localization pattern of major ND10 proteins in THP-1 monocytes; the percentages given at the right of panel a (subpanels e–h and i–l) indicate the fraction of cells (based on the analysis of approx. 70 individual cells) showing the respective colocalization pattern; (**b**) Subnuclear localization pattern of the major ND10 proteins in THP-1 derived macrophages. The following antibodies were used for staining of ND10 components: Anti-PML polyclonal antibody H238 (a, subpanel b, f and j; b, subpanel b and f), anti-Sp100 from Abnova (a and b, subpanel c), anti-hDaxx polyclonal antibody C20 (a, subpanel g and k; b, subpanel g). Nuclei were counterstained with DAPI (4′,6-diamidino-2-phenylindole) (a, subpanel a, e and i; b, subpanel a and e); (**c**) Detection of PML, hDaxx and Sp100 expression pattern in THP-1 monocytes compared to THP-1 derived macrophages by Western blotting. The upper panel shows hDaxx expression levels in both cell types assessed by staining with an anti-hDaxx rabbit monoclonal antibody. The middle panels show detection of various SUMOylated as well as non-SUMOylated PML as well as Sp100 isoforms using anti-PML antibodies A301-167A/A301-168A and anti-Sp100 rabbit serum GH3, respectively. In the lower panel, detection of β-actin was included as an internal loading control.

In order to assess if the major ND10 factors PML, Sp100 and hDaxx are involved in the regulation of the HCMV MIEP in undifferentiated THP-1 monocytes, we wished to analyze HCMV replication in the absence of individual ND10 proteins by generating cells with a stable knockdown of these factors. However, before siRNA-mediated knockdown experiments were performed, we wanted to confirm that THP-1 monocytes contain genuine ND10 structures. Thus, the localization and expression pattern of PML, hDaxx and Sp100 in these cells was analyzed (Figure 1a,c). Indirect immunofluorescence staining revealed the typical colocalization of PML and Sp100 in punctate structures in all cells examined (Figure 1a, subpanels b and c). However, hDaxx localization was not homogenous in all cells since 56% of the cells showed a colocalization of PML and hDaxx in punctate domains (Figure 1a, subpanels f and g), whereas 44% of the cells exhibited a predominantly diffuse, microdispersed localization of hDaxx throughout the nucleus with little PML association (Figure 1a, subpanel k). This is consistent with previous observations for hDaxx localization in CD34+ cells [31]. In parallel, the endogenous level of ND10 proteins was also determined in THP-1 derived macrophages (Figure 1b), which were generated from THP-1 monocytes by the addition of PMA for 48h. Indirect immunofluorescence analysis of these differentiated cells showed the typical association of PML, hDaxx and Sp100 in punctate structures constituting ND10s (Figure 1b). Thus, the localization pattern of the major ND10 structures in THP-1 derived macrophages differs slightly from the pattern in THP-1 monocytes suggesting that differentiation of cells is linked to an enhanced colocalization of PML, hDaxx and Sp100 and the formation of ND10s. In addition, detection of the major ND10 proteins in THP-1 monocytes compared to THP-1 derived macrophages by Western blotting revealed a similar expression pattern of hDaxx and several PML isoforms in both cell types (Figure 1c, upper and middle panel, compare lanes 1 and 2). However, differences in the Sp100 expression pattern could be determined (Figure 1c, third panel). Whereas anti-Sp100 antibody GH3 detected a major signal for non-SUMOylated Sp100 in THP-1 monocytes (Figure 1c, third panel, lane 1), the overall expression level of Sp100 was reduced in differentiated THP-1 cells. This was mainly due to a decrease in the abundance of the non-SUMOylated isoform of Sp100 (Figure 1c, third panel, lane 2).

### 3.2. Generation of THP-1 Cells with a Stable Knockdown of PML, hDaxx or Sp100

**Figure 2:**
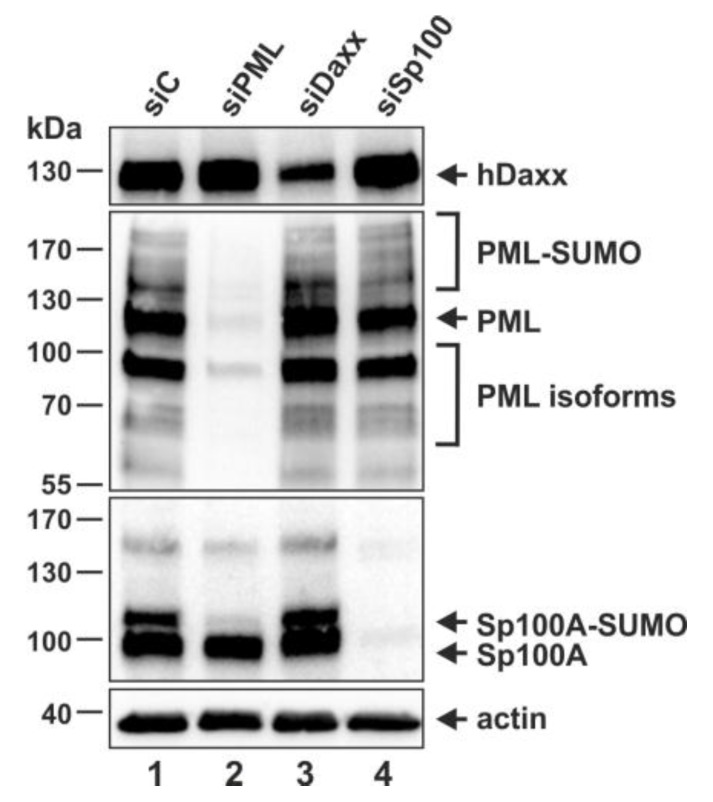
Detection of endogenous hDaxx, PML and Sp100 by Western blotting using cell lysates derived from THP-1 monocytes, which were transduced with the respective shRNA expression vectors as indicated. The upper panel shows hDaxx protein levels detected with an anti-hDaxx rabbit monoclonal antibody. The middle panels show staining of various SUMOylated and non-SUMOylated isoforms of PML using the rabbit polyclonal anti-PML antibodies A301-167A/A301-168A and the SUMOylated as well as non-SUMOylated isoforms of Sp100 with anti-Sp100 rabbit serum GH3. In the lower panel detection of β-actin served as an internal loading control. Lanes: 1, siC control cells; 2, siPML cells; 3, siDaxx cells; 4, siSp100 cells. Quantification of the reduction of protein expression via densitometry revealed a knockdown efficacy of 96% for siPML cells, of 68% for siDaxx cells and of 94% for siSp100 cells, respectively.

Having confirmed the presence of the ND10 proteins PML, hDaxx and Sp100 in THP-1 monocytes by Western blotting and indirect immunofluorescence analysis, we applied an shRNA-directed approach to generate THP-1 monocytes devoid of PML, hDaxx or Sp100. PML, hDaxx and Sp100 shRNA sequences were cloned into the lentiviral expression vector pLVX-shRNA1 followed by production of lentiviral supernatants. Next, THP-1 monocytes were transduced with lentiviruses expressing the respective shRNAs and finally, puromycin-resistant cells were selected. As controls, THP-1 monocytes containing integrated copies of a functionally inactive shRNA sequence (siC cells) were established. The knockdown of PML, hDaxx and Sp100 was assessed by Western blot analysis. Stable expression of an shRNA targeting PML resulted in an extensive depletion of several SUMOylated and non-SUMOylated isoforms of PML (Figure 2, second panel, lane 2). As previously reported for human fibroblast, the knockdown of PML also affected the SUMOylated isoforms of Sp100 (Figure 2, third panel, lane 2) [47]. HDaxx expression was substantially reduced in the generated siDaxx cells (Figure 2, upper panel, lane 3) compared to hDaxx protein levels in the corresponding control cell line siC and in siPML and siSp100 cells (Figure 2, upper panel, lanes 1, 2 and 4). Furthermore, expression of all Sp100 isoforms was completely downregulated in the generated siSp100 THP-1 monocytes (Figure 2, third panel, lane 4), as detected with the anti-Sp100 rabbit serum GH3. In summary, this verified the generation of THP-1 monocytes with a stable knockdown of PML, hDaxx or Sp100.

### 3.3. Analysis of the Role of PML, hDaxx or Sp100 for the Differentiation of THP-1 Monocytes

**Figure 3:**
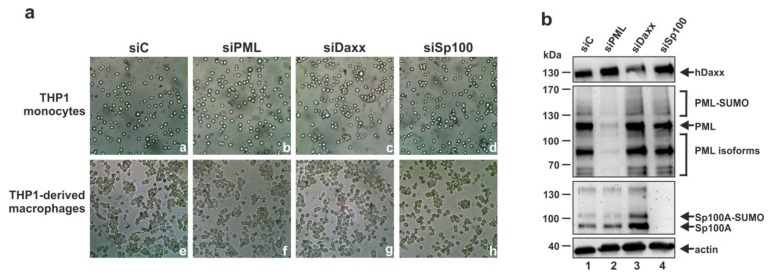
Depletion of PML, hDaxx or Sp100 does not prevent differentiation of THP-1 monocytes to THP-1 derived macrophages. (**a**) THP-1 monocytes were treated with PMA for 48 h followed by comparison of the morphology of THP-1 derived macrophages and THP-1 monocytes by transmitted light microscopy; (**b**) Verification of the knockdown of PML, hDaxx and Sp100 after differentiation of THP-1 monocytes. Cell lysates derived from the respective THP-1-derived macrophages were analyzed by Western blotting for PML, hDaxx and Sp100 expression. Upper panel: Staining of hDaxx protein levels of siC- (lane 1), siPML- (lane 2), siDaxx- (lane 3) and siSp100-transduced cells (lane 4) using a rabbit monoclonal anti-hDaxx antibody. Middle panels: Detection of various SUMOylated and non-SUMOylated isoforms of PML and Sp100 with the rabbit polyclonal anti-PML antibodies A301-167A + A301-168A and the anti-Sp100 rabbit serum GH3, respectively. Lower panel: β-actin detection was included as internal loading control.

Since the results of previous studies suggested that PML may be critical for the differentiation of myeloid progenitor cells to macrophages [48], it was important to investigate whether the knockdown of PML, hDaxx or Sp100 affects the differentiation of THP-1 monocytes to THP-1 derived macrophages. For this, the generated siC, siPML, siDaxx and siSp100 cells were treated with PMA for 48 h to induce terminal differentiation. Generally, effective differentiation is characterized by adherence of the cells and by morphological changes towards a macrophage-like phenotype. Whereas undifferentiated THP-1 cells exhibited the morphology of non-adherent cells (Figure 3a, subpanels a–d), treatment of the cells with PMA resulted in the adherence of all generated knockdown and control cell lines (Figure 3a, subpanels e–h) implying that loss of PML, hDaxx or Sp100 does not prevent differentiation of THP-1 monocytes to THP-1 derived macrophages. Furthermore, the knockdown of PML, hDaxx and Sp100 was also verified in the THP-1 derived macrophages by Western blotting (Figure 3b), ensuring that the knockdown was maintained during differentiation. Taken together, this experiment clearly showed that PML, hDaxx or Sp100 are not involved in the differentiation of THP-1 monocytes to THP-1 derived macrophages.

### 3.4. Construction and Characterization of Recombinant Cytomegaloviruses Expressing the Major Immediate-Early Proteins IE1 or IE2 in Fusion with Autofluorescent Proteins

**Figure 4:**
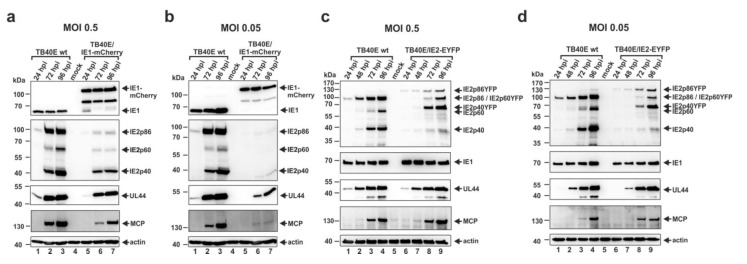
Comparative analysis of viral protein accumulation after infection with wt TB40E and the recombinant viruses TB40E/IE1-mCherry and TB40E/IE2-EYFP. HFFs were infected in parallel with wt TB40E and TB40E/IE1-mCherry (**a** and **b**) or TB40E/IE2-EYFP (**c** and **d**) at an MOI of 0.5 (**a** and **c**) or 0.05 (**b** and **d**). Cell lysates were prepared at the indicated times after virus inoculation (24, 48, 72 and 96 hpi) and subjected to Western blot analyses. The protein levels of the viral immediate-early (IE) proteins IE1 and IE2, the early-late (E) protein UL44 as well as the true late (L) protein MCP were analyzed using specific antibodies against the respective proteins. Detection of β–actin served as an internal loading control.

In order to facilitate the monitoring of HCMV replication and reactivation in THP-1 monocytes we decided to construct recombinant cytomegaloviruses, based on the endotheliotropic strain TB40E, expressing the major immediate-early proteins IE1 and IE2 in fusion with autofluorescent proteins. Thus, the TB40E-based HCMV bacterial artificial chromosome (BAC) TB40E-Bac4 was manipulated in order to fuse the coding sequences of the autofluorescent proteins mCherry or EYFP to the C-terminus of the immediate-early protein IE1 or IE2, respectively [49]. Next, distinct PCR reactions, nucleotide sequence analyses and restriction fragment length polymorphism analyses (RFLP) revealed the correct BAC recombination (data not shown). After reconstitution of infectious viruses, termed TB40E/IE1-mCherry and TB40E/IE2-EYFP, it was necessary to characterize the replication of these recombinant viruses. To address this issue, the viral protein expression kinetics of TB40E/IE1-mCherry and TB40E/IE2-EYFP were compared to that of wt TB40E. HFFs were infected in parallel with the respective viruses at MOIs of 0.5 and 0.05 and at different times postinfection cell lysates were prepared. The accumulation of viral proteins was monitored via immunoblotting with antibodies directed against viral immediate-early (IE1, IE2 isoforms IE2p86, IE2p60, IE2p40), early-late (UL44) and true late (MCP) proteins (Figure 4a–d). The immediate-early proteins IE1 and IE2 exhibited an increased molecular mass in cells infected with TB40E/IE1-mCherry or TB40E/IE2-EYFP, respectively, in comparison to cells infected with wt TB40E (Figure 4a–d, upper panels), which indicates the successful fusion of IE1 to mCherry and of IE2 to EYFP. Whereas the expression levels of IE1 were similar in cells infected with wt TB40E and TB40E/IE1-mCherry, staining of IE2 showed a severely reduced expression of all three isoforms of the respective protein in cells infected with the recombinant virus TB40E/IE1-mCherry (Figure 4a,b). As a consequence of the decreased expression level of IE2 in the TB40E/IE1-mCherry-infected cells, a considerable delay in early (UL44) and late (MCP) protein expression could be detected (Figure 4a,b, third and fourth panel). The diminished abundances of IE2, UL44 and MCP in cells infected with the recombinant virus could be detected under high MOI conditions, but were even more pronounced under circumstances of a low MOI (Figure 4a,b). These results suggest that fusion of mCherry to IE1 negatively affects the expression of IE2 which might be due to splice inhibition since IE1 and IE2 are expressed by alternative splicing from a single mRNA species consisting of five exons [50,51]. In contrast, the protein expression kinetics of TB40E/IE2-EYFP was comparable to that of wildtype virus, suggesting that TB40E/IE2-EYFP could serve as a sensitive tool to analyze viral gene expression in THP-1 cells via flow cytometry.

### 3.5. Infection of Non-Differentiated and Differentiated THP-1 Cells with Recombinant Virus TB40E/IE2-EYFP

**Figure 5:**
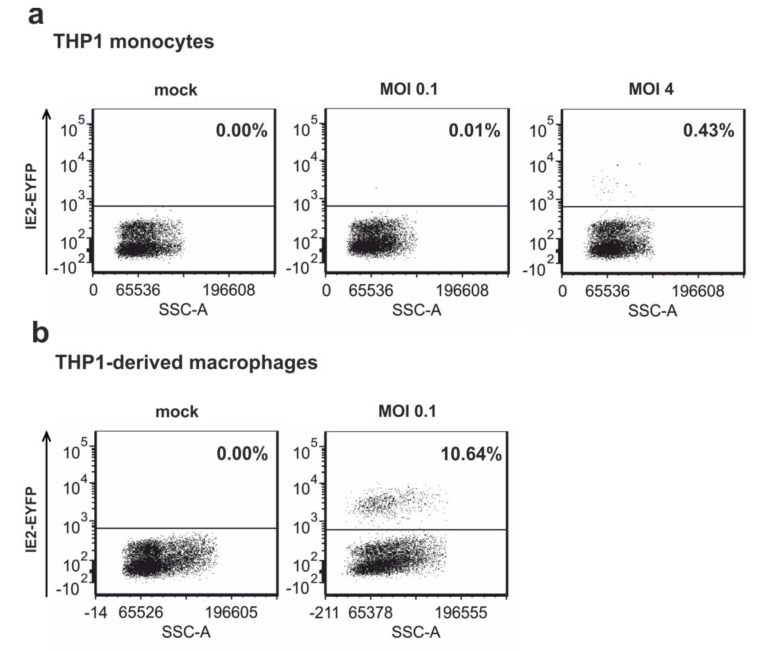
HCMV infection of THP-1 cells results in a quiescent state with almost no detectable IE gene expression. (**a**) THP-1 monocytes were infected with TB40E/IE2-EYFP at an MOI of 0.1 or 4. At 24 hpi, IE2-EYFP positive cells were measured by flow cytometry; (**b**) THP-1 monocytes were differentiated to THP-1 derived macrophages by the addition of PMA for 48 h followed by infection of the obtained differentiated THP-1 cells with TB40E/IE2-EYFP at an MOI of 0.1. At 24 hpi, IE2-positive cells were detected by flow cytometry measuring EYFP autofluorescence.

To investigate whether the recombinant HCMV TB40E/IE2-EYFP could serve as a sensitive tool to monitor HCMV gene expression, THP-1 monocytes were infected with this virus under low (MOI 0.1) and high (MOI 4) MOI conditions. At 24 hpi, flow cytometry analysis was performed in order to quantify the number of IE2 expressing THP-1 monocytes. As expected, infection of THP-1 monocytes at an MOI of 0.1 resulted in little IE2-EYFP expression since only one positive out of 10,000 cells could be detected by flow cytometry analysis (Figure 5a, middle panel). In addition, using high MOI conditions revealed IE gene expression in only 0.43% of the infected cells (Figure 5a, right panel) implying that infection of THP-1 monocytes with HCMV results in a latent-like state. In contrast, THP-1 monocytes, which were differentiated to THP-1-derived macrophages prior to HCMV infection, were permissive for IE gene expression since infection of these cells at an MOI of 0.1 was sufficient to obtain IE2-EYFP expression in 10.64% of the analyzed cells (Figure 5b, right panel). Effective differentiation of THP-1 cells induced by the addition of PMA to the cells for 48 h was verified by monitoring the morphology of the cells (data not shown). In summary, this monocytic cell line enabled us to study if ND10 proteins are involved in the differentiation-dependent regulation of IE gene expression since significant viral IE gene expression could only be observed upon cellular differentiation.

### 3.6. Depletion of the Major ND10 Proteins PML, hDaxx or Sp100 Does Not Affect the Establishment of HCMV Latency in Undifferentiated THP-1 Monocytes

**Figure 6:**
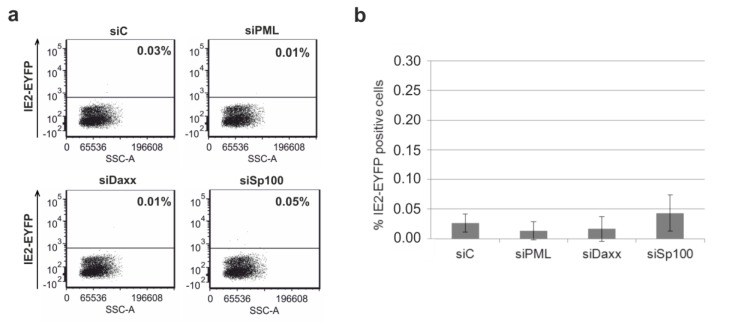
Knockdown of PML, hDaxx or Sp100 in THP-1 monocytes does not permit HCMV IE gene expression. (**a**) Equal numbers of siC, siPML, siDaxx and siSp100 THP-1 cells were infected with TB40E/IE2-EYFP at an MOI of 0.1. EYFP expression was measured at 24 hpi by flow cytometry; (**b**) Mean values of three individual infection experiments according to (**a**). Statistical analyses using the paired t test for siC and siPML, siDaxx and siSp100, respectively, revealed no significant differences between knockdown and control cells siC.

The availability of THP-1 monocytes in which the protein levels of the respective ND10 proteins PML, hDaxx and Sp100 were highly suppressed enabled us to approach the controversially discussed involvement of the major ND10 proteins in the regulation of IE gene expression in undifferentiated cells. As previous studies suggested that ND10 proteins exhibit their repressive function only after infection of cells at low MOI, we first infected knockdown and control cells with TB40E/IE2-EYFP at an MOI of 0.1 [52]. Twenty-four hours later, cells were harvested and examined by flow cytometry for IE2-EYFP expression (Figure 6). As depicted in Figure 6, knockdown of the individual ND10 proteins was not associated with a significant enhancement of IE2-EYFP expression upon HCMV infection at an MOI of 0.1.

**Figure 7:**
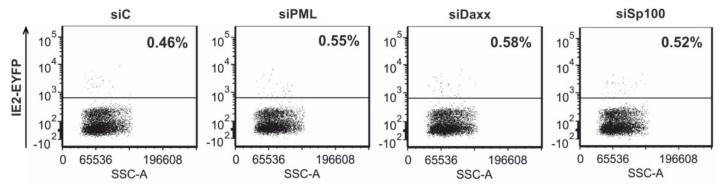
Similar numbers of IE2-EYFP positive cells in PML-kd, hDaxx-kd, Sp100-kd and control THP-1 monocytes after infection of these cells with TB40E/IE2-EYFP at an MOI of 4. At 24 hpi, cells were harvested and IE2-EYFP expression was analyzed by flow cytometry.

As Saffert and Kalejta observed increased IE gene expression in THP-1 cells devoid of hDaxx upon HCMV infection at an MOI of 3 [30] and as high MOIs are used by many groups to establish latency *in vitro*, we also infected control and knockdown THP-1 cells with TB40E/IE2-EYFP at an MOI of 4. Detection of IE gene expression in the individual cell lines was achieved by measuring EYFP autofluorescence at 24 hpi using flow cytometry analysis. However, infection under high MOI conditions did not reveal any significant differences in the numbers of IE2-EYFP positive cells when comparing siC with siPML, siDaxx or siSp100 cells, respectively (Figure 7). In summary, these results suggest that neither PML, hDaxx nor Sp100 are involved in the regulation of IE gene expression in undifferentiated cells and that only differentiation of the cells enables IE gene expression.

### 3.7. Analysis of HCMV IE Gene Expression after Differentiation of PML, hDaxx or Sp100 Knockdown Cells to THP-1 Derived Macrophages

**Figure 8:**
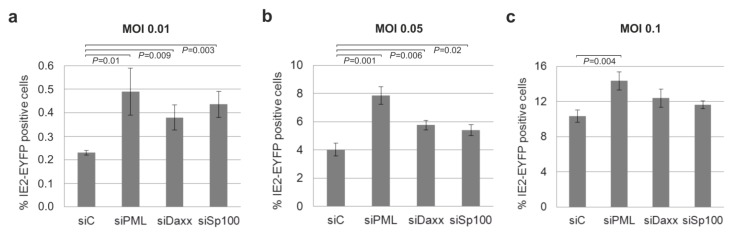
Enhancement of HCMV IE gene expression in the absence of PML, hDaxx or Sp100 under low MOI conditions. THP-1 monocytes, retrovirally transduced as indicated, were differentiated to THP-1-derived macrophages by the addition of PMA for 72 h. Next, these cells were infected with TB40E/IE2-EYFP at an MOI of 0.01 (**a**), 0.05 (**b**) or 0.1 (**c**). At 24 hpi, cells were harvested and examined by flow cytometry for EYFP expression. Each infection was performed in triplicate and the standard deviations are depicted by error bars. Statistical significance (*p*-values) of the difference between control cells and knockdown cells was calculated using Student’s unpaired two-tailed *t*-test.

Having detected no enhancement of IE gene expression in undifferentiated THP-1 cells after depletion of PML, hDaxx, or Sp100, we next wanted to investigate if these cellular proteins play a role for HCMV replication in THP-1 derived macrophages which are permissive for HCMV. Therefore, the individual knockdown cell lines were differentiated to THP-1 derived macrophages by the addition of PMA for 72 h followed by infection of the cells with TB40E/IE2-EYFP at an MOI of 0.01 (Figure 8a), 0.05 (Figure 8b) and 0.1 (Figure 8c). At 24 hpi, IE2-EYFP positive cells were measured by flow cytometry analysis. This experiment revealed significantly more IE2-EYFP positive cells in the absence of PML, hDaxx and Sp100 compared to control cells siC upon infection at an MOI of 0.01 (Figure 8a) and 0.05 (Figure 8b). The effect of more cells initiating the lytic replication cycle in the absence of hDaxx or Sp100 was dependent on the MOI and could only be detected at an MOI of 0.01 (Figure 8a) and 0.05 (Figure 8b). At an MOI of 0.1 (Figure 8c), no significant difference between control cells siC and siDaxx or siSp100, respectively, could be determined by flow cytometry analysis. Interestingly, in contrast to infection of siDaxx and siSp100 cells, infection of siPML cells resulted in significantly more cells expressing IE2-EYFP compared to control cells siC at all three indicated MOIs (Figure 8). In conclusion, these results propose that depletion of PML, hDaxx or Sp100 in THP-1-derived macrophages facilitates the initiation of HCMV IE gene expression, whereas no involvement of these major ND10 proteins in the regulation of HCMV replication in THP-1 monocytes could be detected.

### 3.8. Knockdown of PML, hDaxx or Sp100 Facilitates HCMV Reactivation from Latency

**Figure 9:**
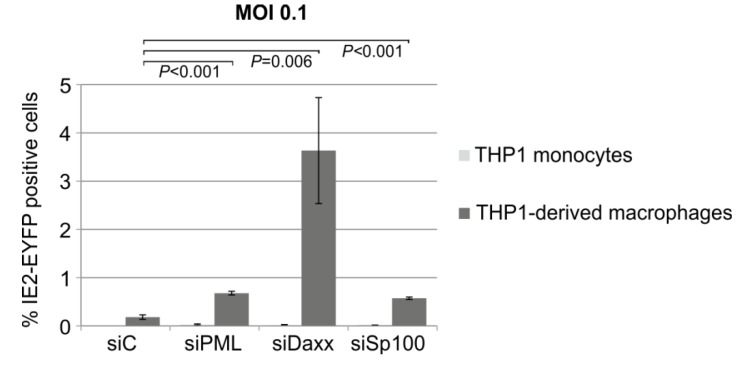
Depletion of PML, hDaxx or Sp100 affects the reactivation efficacy of latent HCMV. THP-1 monocytes, retrovirally transduced as indicated, were infected with TB40E/IE2-EYFP at an MOI of 0.1 and cultured for five days. Thereafter, aliquots of the latently infected THP-1 monocytes were fixed. The remainder of the cells were differentiated to THP-1-derived macrophages by the addition of PMA for 72 h. Then, cells were harvested and THP-1 monocytes as well as macrophages were examined by flow cytometry for EYFP expression. Each infection was performed in triplicate and the standard deviations are depicted by error bars. Statistical significance (*p*-values) of the difference between control cells and knockdown cells was calculated using Student’s unpaired two-tailed *t*-test.

Since infection of already differentiated THP-1 cells revealed differences between PML-, Sp100 or hDaxx knockdown cells compared to control cells concerning the initiation of IE gene expression, we asked the question whether the depletion of ND10 proteins might affect the dynamic process of HCMV reactivation from latency. In order to address this question, THP-1 control and knockdown cells were infected with TB40E/IE2-EYFP at an MOI of 0.1. Five days post-infection aliquots of the infected cells were fixed for a subsequent analysis of IE2-EYFP positive cells in the absence of cellular differentiation. The remaining cells were treated with PMA in order to induce cellular differentiation and, consequently, reactivation of latent HCMV. Three days later cells were harvested and flow cytometry was performed with both differentiated and non-differentiated cells to quantify IE2-EYFP positive cells. This experiment revealed that the knockdown of PML and Sp100 resulted in an approximately threefold increased reactivation efficacy of HCMV compared to the control cells (Figure 9). Interestingly, in hDaxx depleted cells an approximately 20-fold higher number of IE2-EYFP positive cells compared to the control cells could be observed (Figure 9, compare siC and siDaxx). Thus, ND10 proteins seem not to be critical determinants of the establishment of a quiescent state in THP-1 cells but contribute to the restriction of lytic HCMV infection upon differentiation-dependent reactivation of the virus.

## 4. Discussion

Previous studies have demonstrated that the ND10-mediated transcriptional repression of HCMV IE gene expression upon infection of permissive cell types is, at least in part, mediated by regulation of the chromatin structure around the MIEP [15,16]. Interestingly, chromatin-mediated silencing of the MIEP was also shown to regulate viral IE gene expression in non-permissive cell types thereby controlling latency and reactivation of HCMV [5]. Whereas HCMV infection of undifferentiated NT2 or monocytic cells resulted in an association of the MIEP with markers of transcriptional repression, namely methylated histones and the HP1 protein [53], experimental infection of differentiated NT2 or monocyte-derived macrophages exhibited a transcriptionally active chromatin structure [54]. Furthermore, Reeves *et al.* detected an association of the MIEP with HP1 in infected CD34^+^ cells and differentiation of infected CD34^+^ cells and monocytes to mature DCs led to an accumulation of acetylated histones around the MIEP [55]. In a third experimental setting, infection of CD14^+^ monocytes resulted in an association of the MIEP with tri-methylated histone H3K27, which represents a typical marker for repressive chromatin [56]. Thus, several lines of evidence exist that the chromatin structure of the MIEP is a major determinant of the differentiation-dependent control of HCMV latency and reactivation [5]. Furthermore, previous studies implied that the association of viral genomes with ND10 proteins may regulate HSV-1 and HIV-1 latency [57,58]. Consequently, this study was initiated to address the question whether the repressive function of individual ND10 proteins is of importance for the regulation of HCMV viral transcription in undifferentiated cells and might therefore be critical for the establishment of latency.

As an experimental approach, HCMV replication should be analyzed in the absence of individual ND10 factors by generating monocytic cells with an siRNA-mediated knockdown of the major ND10 components PML, hDaxx and Sp100. Due to the inherent difficulties to genetically manipulate primary human monocytes while maintaining them in an undifferentiated state, we took advantage of the monocytic cell line THP-1 which was used in numerous previous studies as an important *in vitro* model for HCMV latency [30,36,59]. Firstly, these cells were characterized regarding the expression of ND10 proteins and the presence of genuine ND10 accumulations. This was important since the embryonal carcinoma cell line NT2, which has been used by a number of laboratories to study latency possesses abnormal ND10 structures resulting from lower expression of the structure defining protein PML and the complete loss of Sp100 [20,60]. Careful examination of THP-1 monocytes using immunofluorescence and Western blot analysis revealed that PML, hDaxx and Sp100 were all expressed in these cells. Differences could be observed between monocytes and THP-1 derived macrophages concerning the colocalization of hDaxx and PML: While in monocytes approximately 40% of the cells exhibited a predominantly diffuse nuclear distribution of hDaxx, differentiation to macrophages correlated with an enhanced colocalization of hDaxx with PML. Since Western blot analyses did not reveal obvious differences in the SUMOylation pattern of either PML or hDaxx, this enhanced colocalization might be due to other, more subtle posttranslational modifications such as phosphorylation or acetylation [23]. Nevertheless, since all major ND10 proteins were detectable, THP-1 cells were regarded as a suitable model system to address if knockdown of the individual ND10 proteins affects HCMV IE gene expression in non-differentiated cells.

Western blot analysis of the generated PML-, hDaxx- and Sp100-depleted THP-1 cells indicated a significant downregulation of hDaxx and an almost complete loss of PML and Sp100 confirming the specificity of the selected shRNAs. Furthermore, depletion of the individual ND10 proteins in THP-1 monocytes did neither induce nor prevent differentiation of these cells to THP-1 derived macrophages. This was important to analyze since a previous study proposed that PML plays a critical role for the differentiation of myeloid progenitor cells to macrophages [48]. This obviously contradictory result may be related to the fact that murine bone marrow macrophages were used in the latter study, whereas our studies were performed with human THP-1-derived macrophages. Thus, one might speculate that PML protein may exhibit species- and cell-type specific functions concerning its role during cellular differentiation.

In order to be able to detect HCMV IE gene expression in a sensitive manner on a single-cell level, we generated recombinant viruses, termed TB40E/IE1-mCherry and TB40E/IE2-EYFP coding for c-terminally mCherry-tagged IE1 or EYFP-tagged IE2, respectively. Importantly, both viruses are based on the endotheliotropic HCMV strain TB40E and are thus suitable for infection of monocytic cells [49]. Unexpectedly, characterization of the TB40E/IE1-mCherry virus revealed a severe replication defect. Analysis of protein expression kinetics demonstrated that this defect is mainly based on a strongly reduced IE2 protein expression and, consequently, a considerable delay in early and late protein expression. Since a similar replication defect was also observed for a recombinant virus expressing IE1 fused to EYFP (data not shown), we speculate that a c-terminal fusion of IE1 may affect the regulation of alternative splicing of the major immediate-early transcripts leading to a decrease of IE2 expression [51]. In contrast, characterization of the generated TB40E/IE2-EYFP virus displayed a protein expression kinetics comparable to wildtype virus. This virus thus constitutes a useful tool to study HCMV latency and reactivation in monocytic cells by flow cytometry.

One major argument for using THP-1 monocytes as a model for HCMV latency was that several groups were not able to detect IE gene expression in these cells [36,59]. In accordance with this, infection of non-differentiated THP-1 cells with TB40E/IE2-EYFP resulted in almost no detectable IE gene expression under low and even very high MOI conditions. However, differentiation of the cells to THP-1 derived macrophages followed by experimental infection revealed IE gene expression even under low MOI conditions. Since ND10 proteins are part of the intrinsic response mechanism that is responsible for the repression of IE gene expression at early times post infection in fully permissive cells [15,61], one major aim of this work was to analyze if these cellular restriction factors might play a role in the regulation of the viral MIEP in undifferentiated cells. However, dissection of HCMV replication in the generated PML-, hDaxx- and Sp100-depleted THP-1 cells argues against an essential role of ND10 proteins for the establishment of HCMV latency since no enhanced IE gene expression could be detected in the absence of the individual proteins. It is important to emphasize that similar results were obtained using both low and high MOI conditions. This indicates that ND10 proteins either play no role for MIEP regulation in undifferentiated THP-1 cells or that the establishment of latency involves many different factors and ND10 proteins are not the determining ones. This assumption is further supported by findings of the Sinclair group which also argue for little involvement of hDaxx in the differentiation-dependent regulation of HCMV IE gene expression [33]. In this study, NT2 cells were used as a model system and infection was performed using the HCMV strain Toledo and low MOI conditions. In contrast, several other studies used high MOI conditions which are usually applied in latency models [30,31,32,62]. Most importantly, studies of the Kalejta group provide evidence that hDaxx silences the viral MIEP because virion-delivered pp71 fails to reach the nucleus and to inactivate the hDaxx protein in non-permissive NT2, THP-1 and CD34+ cells [30,31]. However, it is noteworthy that only IE gene expression of laboratory-adapted strains, but not of clinical strains, could be rescued by knockdown of hDaxx [31]. In order to explain this discrepancy, the authors proposed that HCMV uses at least three different mechanisms to restrict viral IE gene expression during the establishment of latency: The low activity of the MIEP in non-permissive cells, the antiviral activity of the subnuclear structure ND10, and the existence of an unidentified viral function that is characteristic of only low-passage viral strains [31]. Thus, it is tempting to speculate that the reason for the opposing results obtained by the Kalejta group in contrast to the Sinclair group and our group might be due to the use of different HCMV strains. However, studies performed in our group using the laboratory-adapted strain AD169 and high MOI conditions, so far, did not rescue IE gene expression in the generated PML-, hDaxx- and Sp100-kd THP-1 cells (data not shown).

In addition, this study was extended by analyzing whether depletion of PML, hDaxx and Sp100 enhances IE gene expression in THP-1-derived macrophages. Experiments using low MOI conditions revealed significantly more cells that initiate the lytic replication program in cells deficient for PML, hDaxx or Sp100. As also observed in previous studies, this enhancing effect exhibited a strong MOI dependence, which is explained by the fact that restriction by ND10 proteins is amenable to antagonization by viral factors like the tegument protein pp71 and the immediate early protein IE1 [52,61]. Nevertheless, these findings show that PML, hDaxx and Sp100 also exhibit an intrinsic repressor function against HCMV in THP-1-derived macrophages. Furthermore, we investigated whether the knockdown of PML, hDaxx or Sp100 might affect the dynamic process of HCMV reactivation from latency. While the depletion of all major ND10 proteins was able to increase the efficacy of viral reactivation, the approximately 20-fold enhancement by hDaxx depletion by far exceeded the effects observed for PML- or Sp100-depleted cells. This dominant effect of hDaxx on the reactivation efficacy might be explained by the fact that the tegument protein pp71 is missing during reactivation, while IE1, which antagonizes PML, is expressed as reactivation proceeds. In summary, by investigating the *in vitro* latency model of THP-1 cells, this study provides evidence that PML, hDaxx and Sp100 do not act as critical determinants for the establishment of a quiescent state after infection of monocytes with human cytomegalovirus. However, they do contribute to the intrinsic immune response of THP-1 derived macrophages and affect the efficacy of differentiation-induced reactivation of HCMV from latency.
